# The Role of Targeted Rehabilitation in Enhancing Recovery After Total Hip Arthroplasty with Anterior Minimally Invasive Surgery Approach: A Retrospective Pilot Study

**DOI:** 10.3390/jfmk11040409

**Published:** 2026-10-05

**Authors:** Riccardo Giorgino, Ricardo Ciliberto, Mileva Sara Roumenova, Matteo Del Re, Nicolò Rossi, Corrado Sosio, Paolo Sirtori, Giuseppe M. Peretti, Laura Mangiavini

**Affiliations:** 1 IRCCS Ospedale Galeazzi-Sant’Ambrogio, 20157 Milan, Italy; 2 Department of Precision and Regenerative Medicine and Ionian Area (DiMePreJ), University of Bari Aldo Moro, 70121 Bari, Italy; 3 Faculty of Medicine and Surgery, University of Milan, 20122 Milan, Italy; 4 Department of Biomedical Sciences for Health, University of Milan, 00133 Milan, Italy

**Keywords:** total hip arthroplasty, coxarthrosis, physical therapy, muscle strength, functional recovery, postoperative care

## Abstract

**Background**: Hip osteoarthritis is a highly prevalent degenerative condition and a leading indication for total hip arthroplasty (THA). As anterior approaches have gained popularity, optimizing postoperative rehabilitation according to the muscles involved in recovery has become increasingly relevant. However, evidence regarding muscle-specific rehabilitation following the Anterior Minimally Invasive Surgery (AMIS) approach remains limited. **Objectives**: This study aimed to evaluate whether rehabilitation protocols specifically targeting the hip flexors or abductors could improve postoperative functional recovery compared with standard rehabilitation following AMIS THA. **Methods**: This retrospective single-center study included 29 patients aged 50–80 years with BMI < 30 kg/m^2^ undergoing unilateral THA through the AMIS approach. The cohort included 14 men and 15 women. Patients were non-randomly allocated according to clinical and organizational factors to standard rehabilitation (ST; n = 10), a hip flexor-focused protocol (SP1; n = 10), or a hip abductor-focused protocol (SP2; n = 9). Postoperative recovery was evaluated using clinical, functional, and patient-reported outcome measures. Rehabilitation was supervised by physiotherapists. Outcomes included goniometric hip range of motion, dynamometer-measured hip flexor and abductor strength, Timed Up and Go (TUG), Visual Analogue Scale (VAS), Harris Hip Score (HHS), Hip Disability and Osteoarthritis Outcome Score (HOOS), and SF-12. Longitudinal outcomes were analyzed using two-factor models assessing time, rehabilitation group, and their interaction, with post hoc comparisons where appropriate. Statistical significance was set at *p* < 0.05. **Results**: Of the 29 initially enrolled patients, 26 completed the study assessments and were included in the final analysis (ST, n = 9; SP1, n = 10; SP2, n = 7). Significant improvements over time were observed for hip flexion and internal rotation ROM, abductor strength, TUG, VAS, HOOS, and HHS. No consistent superiority of either targeted protocol over standard rehabilitation was identified. Postoperative improvements in hip range of motion, muscle strength, mobility, pain, and functional performance were observed over time across the study cohort. Although no protocol demonstrated statistically significant superiority in range of motion or functional outcomes, all three groups showed postoperative improvement over time following AMIS THA. **Conclusions**: All rehabilitation groups improved during postoperative recovery, but neither the flexor- nor abductor-focused protocol demonstrated superiority over standard rehabilitation. Given the small sample size and non-randomized design, these findings are preliminary and hypothesis-generating and require confirmation in adequately powered randomized studies. Because the targeted protocols also involved a greater overall number of prescribed exercises, the present design cannot distinguish the effects of muscle-specific targeting from those of greater exercise exposure.

## 1. Introduction

The degenerative arthritic pathology of the hip, or coxarthrosis, is an extremely-common condition affecting approximately 10% of the adult population, with 50% of cases being symptomatic [1,2]. Hip osteoarthritis represents a growing clinical burden, particularly in aging populations, with increasing demand for surgical intervention in younger and more active patients. Additionally, coxarthrosis represents the most frequent cause of disability in the adult-elderly population [3,4] and can initially be managed conservatively; however, definitive treatment is predominantly surgical [5]. In advanced stages of the pathology, hip prosthetic replacement represents the gold standard therapeutic approach and one of the major successes of modern orthopedic surgery [6,7]. In recent years, indications for hip prosthetic replacement surgery have expanded, the average age of patients undergoing the procedure has decreased, and consequently, patient expectations have significantly increased [8]. Hip osteoarthritis is characterized by progressive degeneration of articular cartilage accompanied by alterations of the subchondral bone, synovial inflammation, osteophyte formation, and changes in periarticular tissues. These structural and biological alterations contribute to pain, joint stiffness, loss of range of motion, muscle weakness, and progressive functional impairment [1,2]. Before surgical treatment is considered, management generally includes patient education, activity modification, weight management when appropriate, structured therapeutic exercise and physiotherapy, together with pharmacological pain management according to individual patient characteristics. Total hip arthroplasty is generally considered when symptoms and functional limitations remain substantial despite appropriate conservative management [1,6,7].

There are several surgical techniques with different approaches to perform this procedure, including the anterior approach. In recent years, minimally invasive surgical approaches, particularly the direct anterior approach, have gained popularity due to potential advantages such as reduced soft tissue damage, faster recovery, and earlier mobilization [9,10]. Within the anterior approach technique, the Anterior Minimally Invasive Surgery (AMIS) is a further type of minimally invasive anterior approach [11,12]. In general, each surgical approach entails specific tissue trauma; in the case of the anterior approach, there is involvement of the flexor musculature due to intraoperative spreading of the sartorius and rectus femoris, and laterally, of the tensor fasciae latae [11,13]. Thus, it can be asserted that the muscle groups most affected by this type of surgical approach are the hip flexors and abductors (which may already be weak preoperatively).

Postoperative rehabilitation plays a crucial role in optimizing functional recovery following total hip arthroplasty. Early mobilization and structured physiotherapy programs have been shown to improve muscle strength, gait performance, and patient-reported outcomes while also reducing complications such as joint stiffness and prolonged disability [13]. However, rehabilitation protocols after THA vary considerably in terms of exercise type, intensity, and timing, and there is currently no consensus on the most effective strategy. In particular, limited evidence is available regarding the potential benefits of muscle-specific rehabilitation protocols tailored to the surgical approach used.

Therefore, it is essential to establish a rehabilitation program tailored to the surgical procedures adopted. Joint prosthetic replacement surgery cannot and should not be limited to recreating anatomical relationships but should be accompanied by a rehabilitation program aimed at restoring physiological gait patterns [14,15,16].

Early postoperative rehabilitation, in addition to increasing patient autonomy and safety in activities of daily living (ADL) and ambulation with assistive devices, includes an exercise program that should be performed at home or in rehabilitation facilities. It has been widely demonstrated that physical exercise yields long-term benefits in patients undergoing hip prosthetic replacement surgery [16]. Early mobilization represents the gold standard for achieving functional mobility after total hip arthroplasty (THA), as well as for reducing hospital stay duration and alleviating pain symptoms [16,17,18,19].

Moreover, increased strength and improved ability to bear weight on the operated limb could reduce the risk of falls [20]. Nevertheless, there are no studies in the literature demonstrating, in relation to surgical approach, the efficacy of one physiotherapy pathway over another. Rehabilitation protocols after hip arthroplasty vary in the types of exercises and the timing of their administration in the postoperative period [18,21].

Although early mobilization and structured rehabilitation are established components of recovery after THA, considerable heterogeneity remains regarding exercise selection, intensity, timing, and progression [18,21]. Current evidence supports postoperative exercise for improving function and muscle recovery, but comparatively little information is available on whether rehabilitation should be specifically tailored to the muscle groups most relevant to the surgical approach. This represents a clinically relevant knowledge gap, particularly for minimally invasive anterior approaches, in which early functional recovery is a major objective.

Accordingly, the present study explored whether muscle-specific rehabilitation targeting the hip flexors or abductors could provide additional functional benefits compared with a standard rehabilitation protocol following AMIS THA. We hypothesized that muscle-specific rehabilitation would result in greater postoperative improvement than standard rehabilitation, and more specifically, that flexor-focused rehabilitation would preferentially improve hip flexor strength and related functional outcomes, whereas abductor-focused rehabilitation would preferentially improve abductor strength and mobility-related outcomes.

## 2. Materials and Methods

### 2.1. Study Design and Patient Selection

This retrospective, single-center observational study was conducted at IRCCS Istituto Ortopedico Galeazzi, Milan, Italy. Patients undergoing elective primary total hip arthroplasty through the AMIS approach between April and June 2022 were retrospectively included with clinical and functional outcomes assessed through the first postoperative month. The population comprised 29 subjects. All patients underwent elective total hip arthroplasty with the AMIS technique [11]. All patients were divided into three groups: ten patients in the 1st group (ST), ten in the 2nd group (SP1), and nine in the 3rd group (SP2). Group allocation was not randomized and was determined by clinical practice and organizational factors within the rehabilitation unit (e.g., protocol availability and physiotherapy scheduling). Twenty-nine patients were initially included (ST, n = 10; SP1, n = 10; SP2, n = 9). Three patients did not complete the study assessments, resulting in a final analyzed cohort of 26 patients (ST, n = 9; SP1, n = 10; SP2, n = 7). Reasons for exclusion are reported in Section 3. This was a single-center retrospective observational pilot study based on routinely collected clinical data. The rehabilitation protocols were implemented as part of the clinical rehabilitation pathways available during the study period and were not assigned prospectively for the purposes of the present analysis. Eligible patients and their clinical and functional data were subsequently identified retrospectively and analyzed according to the rehabilitation protocol received.

### 2.2. Eligibility Criteria

Inclusion criteria were the following: age between 50 and 80 years; body mass index (BMI) <30; patients with primary or secondary coxarthrosis due to femoroacetabular impingement (CAM and/or Pincer type), femoral head osteonecrosis, developmental hip dysplasia Crowe I and II; patients undergoing anterior approach total hip arthroplasty with the AMIS technique. Exclusion criteria were the following: hip arthroplasty revisions, developmental hip dysplasia Crowe III–IV; severe congenital or post-traumatic hip morphological alterations; ongoing neurological and oncological conditions; patients with contralateral THA; patients who underwent bilateral THA implantation.

### 2.3. Surgical Procedure and Perioperative Management

All patients underwent primary total hip arthroplasty through the AMIS approach. Procedures were performed by four experienced hip surgeons using Medacta implants (Medacta International, Castel San Pietro, Switzerland) and a dedicated AMIS operating table. Postoperatively, weight bearing was allowed as tolerated from the early postoperative period. Detailed patient-level information regarding perioperative analgesic regimens was not systematically available in the retrospective dataset and was therefore not included in the present analysis.

### 2.4. Rehabilitation Protocols

Each group was assigned a rehabilitation protocol. The control group used the currently implemented rehabilitation protocol (ST) in the Enhanced Recovery After Surgery (ERAS) department of our hospital; the second group followed an experimental rehabilitation protocol (SP1) focusing on targeted strengthening of the hip flexor muscles; the third group followed an experimental protocol (SP2) targeting strengthening of the hip abductor muscles (Table 1). The targeted protocols also included a greater overall number of prescribed exercises than standard rehabilitation, precluding the separation of muscle-targeting effects from differences in exercise volume. Although the protocols primarily differed in targeted muscle groups, the total number of exercises was higher in the experimental groups, which may represent a confounding factor influencing the results. All groups followed rehabilitation programs with the same general postoperative rehabilitation schedule; however, the total exercise volume differed between protocols and the actual session duration was not systematically recorded. The protocols differed in both the muscle groups emphasized and the overall number of prescribed exercises, with a greater exercise volume in the targeted rehabilitation groups than in the standard rehabilitation group. The experimental protocols included 7 exercises targeting the specific muscle groups intended for reinforcement. Therapeutic exercises within each protocol were performed twice daily (morning and afternoon) in the ward under the supervision of a physiotherapist throughout the patient’s hospital stay. Upon discharge, patients were instructed to perform the exercises a minimum of two times daily, taking into account their pain and fatigue levels. Two sets of 10 repetitions were prescribed for each exercise. All strengthening exercises were performed at low to moderate intensity, adapted to each patient’s pain tolerance and functional capacity, without the use of external loads in the early postoperative phase. The progression of exercises was based on clinical improvement, with a gradual increase in range of motion and repetitions as tolerated. During hospitalization, all exercises were supervised by a physiotherapist to ensure correct execution. The standard rehabilitation protocol represented the routine ERAS rehabilitation pathway used at our institution. The two experimental protocols were developed within the rehabilitation unit for the purposes of this study by modifying the standard pathway through additional exercises specifically targeting either the hip flexor or hip abductor musculature. The protocols were therefore institution-based and were not directly reproduced from a previously published protocol.

### 2.5. Clinical and Functional Assessment

All patients were evaluated using clinical and functional scores at various time points to analyze their progression over time (preoperatively, pre-discharge, at two weeks, and one month post-surgery). These scores were instrumental in determining joint functionality, muscular recovery, and quality of life following the patient’s undergone procedure. The parameters and scales utilized were the following: range of motion (ROM); muscle strength; Hip Disability and Osteoarthritis Outcome Score (HOOS) [22]. Hip-related symptoms and function were assessed using the complete 40-item Hip disability and Osteoarthritis Outcome Score (HOOS), comprising five domains: Pain, Symptoms, Activities of Daily Living, Sport and Recreation, and hip-related Quality of Life. For the purposes of the present exploratory analysis, responses across all HOOS items were aggregated and transformed to a 0–100 scale, with higher scores indicating better hip-related status. This aggregate measure was used as a descriptive overall HOOS score and should not be interpreted as one of the standard individual HOOS subscale scores; Harris Hip Score (HHS) (higher HHS scores indicate better hip function) [22]; 12-item Short Form Survey (SF-12) (higher PCS-12 and MCS-12 scores indicate better perceived physical and mental health, respectively) [23]; Visual Analogue Scale (VAS) (VAS ranges from 0 to 10, with higher scores indicating greater pain intensity) [24]; Timed Up and Go (TUG) [25]. Assessment time points were defined as follows: T0, preoperative baseline; T1, before hospital discharge; T2, approximately 14 days postoperatively; and T3, approximately one month postoperatively. Not all outcomes were collected at every time point, as specified for each outcome measure below and in the corresponding figure legends.

Hip ROM measurements were performed using a standard manual universal 360° goniometer (non-electronic), with proper support and the limb positioned anatomically. Clinical and functional assessments were performed by trained physiotherapists with the standardized assessment procedures used in the rehabilitation unit. Outcome assessors were not blinded to group allocation. Each joint movement was measured from a neutral starting position, either corresponding to the anatomical position or to a predefined zero position. In this study, ranges of abduction, adduction, flexion, internal rotation, and external rotation of the joint were measured. Hip extension was not included among the ROM outcomes analyzed in the present study. The peak isometric strength of the hip abductors and flexors was measured using a portable dynamometer (model 01165; Lafayette Instrument Company, Lafayette, IN., USA). Peak force was recorded in Newtons during a 5-s isometric contraction and was not normalized to body mass. Patients were provided with a home rehabilitation diary to record whether the prescribed exercises were performed. However, adherence data were not available with sufficient completeness for reliable quantitative comparison between groups and were therefore not included as an outcome in the present analysis. The study protocol was covered by the original ethical approval (02/07/2015/No. 82/INT/2015), which included observational studies on patients undergoing hip arthroplasty. All patients provided informed consent for participation and data use. No substantial modifications to the study design requiring additional approval were introduced during the study period.

### 2.6. Statistical Analysis

The statistical analyses were conducted using Prism v8.0 software (GraphPad Software, La Jolla, CA, USA). Data distribution was assessed using the Shapiro–Wilk test. Longitudinal outcomes were analyzed using two-way repeated-measures ANOVA, with time representing the within-subject factor and rehabilitation group the between-subject factor, including assessment of the time-by-group interaction. Pairwise comparisons were conducted using the Tukey post hoc test where appropriate. Data are presented in box plots as median, interquartile range, and minimum/maximum values. Statistical significance was set at *p* < 0.05. No a priori sample size calculation was performed due to the retrospective design; therefore, it cannot be established whether the analyzed sample was sufficiently powered to confirm or refute the prespecified hypotheses or to detect clinically meaningful between-group differences. The study should therefore be considered underpowered to detect small-to-moderate between-group differences.

Due to the retrospective design, not all variables were available at each time point for all patients, resulting in some missing data across follow-up assessments. No single primary endpoint or primary time point was prospectively prespecified. Given the retrospective pilot design, all analyses should therefore be considered exploratory. Because of the retrospective nature of the dataset, the exact pattern of missing observations and the outcome-specific number of participants contributing to each longitudinal model could not be reliably reconstructed for all outcomes. No imputation of missing observations was performed. Accordingly, the longitudinal analyses should be considered exploratory.

## 3. Results

Twenty-nine patients were initially enrolled, including 14 men (48.3%) and 15 women (51.7%). Baseline demographic characteristics are reported in Table 2. Three patients did not complete the study, resulting in a final analyzed cohort of 26 patients. One patient in the ST group was excluded after baseline assessment because of a preoperative transient ischemic attack. Two patients in SP2 did not complete follow-up: one withdrew from the study and one developed COVID-19, preventing attendance at subsequent hospital assessments. The final analyzed groups therefore consisted of 9 ST, 10 SP1, and 7 SP2 patients.

The participant flow and reasons for incomplete postoperative assessment are summarized in Figure 1.

The number of observations varied slightly across time points due to missing follow-up data. One patient initially identified for the ST group was excluded after the preoperative baseline assessment because a transient ischemic attack occurred before surgery, preventing progression to THA and postoperative rehabilitation. The patient with TIA was excluded after the baseline assessment and before completing the rehabilitation protocol. In the SP2 group, two patients were excluded: one patient refused to continue participating in the study, and the second patient contracted COVID-19 infection, making it impossible to attend hospital follow-ups.

Baseline demographic and clinical characteristics of the initially enrolled population are summarized in Table 2.

Given the exploratory nature and limited sample size of the study, inferential results are primarily reported as time effects and time-by-group interactions with their corresponding *p*-values. The findings should therefore be interpreted as exploratory rather than as precise estimates of treatment effects.

Hip flexion range of motion significantly increased over time across all groups, indicating progressive postoperative recovery. However, no significant differences were observed between the rehabilitation protocols, suggesting a similar improvement pattern regardless of the intervention. The parameter of flexion range of motion (ROM FLEX) (Figure 2) progressively increased over time (*p* < 0.001), but there was no effect of the group assignment on this change (time*group interaction, *p* = 0.184).

Hip abduction range of motion did not show a statistically significant change over time and no differences were observed between groups, indicating that the rehabilitation protocols did not differentially influence this parameter. The parameter of abduction range of motion (ROM ADB) (Figure 3) did not show significance either over time or based on group assignment (interaction, *p* = 0.821).

Hip adduction range of motion showed a modest but statistically significant improvement over time. However, no between-group differences were detected, suggesting comparable outcomes among the rehabilitation protocols. The parameter of adduction range of motion (ROM ADD) (Figure 4) progressively changed over time (*p* = 0.015), but there was no effect of group assignment on this change (time*group interaction, *p* = 0.832).

Internal rotation range of motion significantly increased over time in all groups, reflecting functional recovery. No significant interaction between time and group was observed. The parameter of internal rotation range of motion (ROM ROI) (Figure 5) progressively increased over time (*p* < 0.001), but there was no effect of group assignment on this change (time*group interaction, *p* = 0.863).

External rotation range of motion did not significantly change over time. Although a difference in mean values between groups was observed, no specific pairwise differences were identified, and this finding should be interpreted cautiously. The parameter of external rotation range of motion (ROM ROE) (Figure 6) did not change over time (*p* = 0.780), and there was no difference in the temporal progression among the three groups (time*group interaction, *p* = 0.607). No significant time-by-group interaction was observed for external rotation (*p* = 0.607), and post hoc pairwise comparisons did not identify significant differences between rehabilitation groups at individual assessment time points.

Hip abductor muscle strength significantly improved over time across all groups. However, no significant differences between groups were detected, indicating that all rehabilitation protocols were similarly effective in enhancing abductor strength. The parameter of abductor muscle strength (FM ABD) (Figure 7) progressively increased over time (*p* = 0.005), but there was no effect of group assignment on this change (time*group interaction, *p* = 0.240).

Hip flexor muscle strength did not show a statistically significant change over time, and no differences between groups were observed, suggesting limited short-term recovery in this parameter. The parameter of flexor muscle strength (FM FLEX) (Figure 8) did not show significance either over time or based on group assignment (interaction, *p* = 0.246).

Timed Up and Go performance significantly improved over time, reflecting enhanced functional mobility in all patients. No differences between rehabilitation groups were observed. The parameter of the Timed Up and Go (TUG) test (Figure 9) significantly decreased over time (*p* = 0.025), but there were no differences in the change based on group assignment (interaction, *p* = 0.339).

Pain levels, as measured by VAS, significantly decreased over time in all groups, indicating postoperative recovery. No significant differences between rehabilitation protocols were detected. The parameter of the Visual Analogue Scale (VAS) (Figure 10) significantly decreased over time (*p* < 0.001), but there were no differences in the change based on group assignment (interaction, *p* = 0.298).

The PCS-12 parameter showed a statistically significant interaction between time and group. However, given the limited sample size and the absence of correction for multiple comparisons, this finding should be interpreted with caution. The parameter PCS-12 (Physical Component Summary) (Figure 11) significantly changed over time in a group-dependent manner (interaction, *p* = 0.014). Although a statistically significant interaction was observed for PCS-12, this finding should be interpreted with extreme caution given the small sample size, multiple comparisons, and absence of correction for Type I error. Therefore, no definitive conclusions can be drawn regarding between-group differences for this parameter. However, the growth over time was significant for the entire sample (*p* < 0.001).

No significant changes over time or differences between groups were observed for the MCS-12 parameter. No differences emerged regarding the parameter MCS-12 (Mental Component Summary) (Figure 12) of the SF-12 scale, neither based on time nor based on group assignment.

HOOS scores significantly improved over time in all groups, reflecting overall functional recovery. No differences between rehabilitation protocols were identified. The parameter of the HOOS questionnaire (Figure 13) progressively increased over time (*p* < 0.001), but there was no effect of group assignment on this change (time*group interaction, *p* = 0.765).

Harris Hip Score significantly improved over time across all groups, with no significant differences between rehabilitation protocols. The parameter of the HHS questionnaire (Figure 14) progressively increased over time (*p* < 0.001), but there was no effect of group assignment on this change (time*group interaction, *p* = 0.350).

## 4. Discussion

The principal finding of this exploratory study was that all three rehabilitation strategies were associated with a favourable postoperative recovery following total hip arthroplasty performed through the AMIS approach. Improvements over time were observed in hip range of motion, mobility, pain, patient-reported function, and several measures of muscle performance. However, neither the flexor-focused nor the abductor-focused protocol demonstrated consistent superiority over standard rehabilitation. Given the limited sample size and retrospective design, these findings should be interpreted within the context of an exploratory retrospective pilot study and should not be considered evidence of equivalence between protocols. Rather, they indicate that no superiority of either targeted protocol could be demonstrated in the present cohort and provide preliminary, hypothesis-generating information for future adequately powered studies. Rather, these preliminary findings warrant further evaluation in adequately powered prospective studies. The optimal rehabilitation strategy following THA remains debated [26]. Early mobilization and progressive strengthening programmes can improve postoperative function and muscle recovery [27,28,29,30], but limited evidence is available regarding rehabilitation protocols tailored to the muscles most relevant to a specific surgical approach [29]. This question may be particularly relevant following an anterior approach, in which the periarticular musculature is preserved through an intermuscular and internervous surgical interval. The present study therefore extends the available literature by exploring whether selective reinforcement of the hip flexors or abductors may provide additional benefits over a standard rehabilitation pathway after AMIS THA. The intensified programmes were implemented during the study period; however, adherence, completed session numbers, and intervention-related adverse events were not systematically quantified. Therefore, no definitive conclusions regarding feasibility or tolerability can be drawn from the present dataset. Postoperative improvements in most clinical and functional outcomes were observed over time across all three groups; however, these changes cannot be attributed specifically to the rehabilitation protocols. Nevertheless, the absence of significant between-group differences in range of motion, HHS, HOOS, TUG performance, and most muscle-strength measures suggests that selectively targeting the hip flexors or abductors did not produce a clearly detectable additional benefit within the follow-up period and sample examined.

Contrary to our initial hypothesis, no consistent group-specific pattern of muscle-strength recovery was identified. Although numerically greater improvements in flexor strength were observed in the SP1 group, these differences did not translate into superior functional outcomes. Similar short-term limitations in strength recovery following THA have been reported by Jensen et al. [31], while Frost et al. described comparable trends following targeted strengthening [32]. In the SP2 group, selective abductor reinforcement did not result in a significant advantage in abductor strength or related functional measures. These findings may indicate that early recovery after THA is influenced by multiple interacting factors—including postoperative inhibition, pain, preoperative muscle status, age, and overall exercise exposure—rather than by selective strengthening of a single muscle group. A significant time-by-group interaction was observed for the physical component score of the SF-12, with numerically higher postoperative values in the abductor-focused SP2 group. This finding is of potential interest because hip abductors play a central role in pelvic stability, gait efficiency, and perceived physical function, and previous studies have suggested that targeted abductor strengthening may improve patient-reported physical health [33]. However, this isolated result should be interpreted cautiously. No correction for multiple comparisons was applied, increasing the possibility of a type I error. Moreover, complete group-stratified baseline characteristics were unavailable, preventing the assessment of potential baseline imbalances between rehabilitation groups. Accordingly, the PCS-12 finding should be regarded as hypothesis-generating rather than evidence of a specific treatment effect. Ambulation improved over time in all three groups, without a clear between-group advantage. Efficient and stable gait is clinically relevant because it supports independence and may reduce the risk of falls, particularly in the context of accelerated rehabilitation and shorter hospital stays [21,26,34,35]. Pain followed a less consistent pattern. Overall, these findings suggest that muscle-specific protocols were tolerated, although transient pain responses during the early postoperative phase merit further investigation. The biological and biomechanical rationale for targeted rehabilitation may be particularly relevant following AMIS THA. The approach uses the anatomical interval between the tensor fasciae latae and sartorius muscles, thereby limiting direct muscle or tendon detachment. MRI-based studies have reported fewer signs of muscle damage, fatty atrophy, and tendon disruption—particularly involving the gluteus medius and minimus—after direct anterior compared with transgluteal approaches [36,37,38]. Preservation of neuromuscular integrity could theoretically facilitate early active rehabilitation and responsiveness to selective strengthening. This interpretation is mechanistic and hypothesis-generating and was not directly tested in the present study. Nevertheless, the present study did not compare different surgical approaches and therefore cannot determine whether the observed recovery patterns were specifically attributable to AMIS. This proposed interaction between surgical approach and muscle-specific rehabilitation should be tested directly in future comparative studies. Regardless of the surgical approach, early mobilization and structured postoperative rehabilitation are key components of functional recovery after THA, improving muscle function and patient-reported outcomes [15,39].

A strength of this study was the multidimensional assessment of postoperative recovery using objective measures of range of motion, muscle strength, and mobility together with validated patient-reported outcome measures. The study also provides preliminary clinical data on two muscle-specific rehabilitation strategies in a field in which evidence remains limited. Furthermore, the inclusion of a standard rehabilitation group allowed the recovery trajectories associated with the experimental protocols to be interpreted within routine postoperative care. Several limitations must, however, be acknowledged. First, the small sample size and subdivision of the cohort into three groups substantially reduced statistical power, limiting the ability to detect small-to-moderate differences and increasing the risks of both type I and type II errors. No a priori sample-size calculation was performed because of the retrospective design. Furthermore, three out of the 29 initially enrolled patients did not complete the study assessments, resulting in a final analyzed cohort of 26 patients with unequal group sizes (ST, n = 9; SP1, n = 10; SP2, n = 7). This attrition further reduced statistical precision, particularly in the smallest group. Second, the lack of randomization introduced a risk of selection bias, while the absence of blinded outcome assessment may have contributed to measurement bias. Moreover, because allocation was non-randomized, differences in baseline characteristics may have influenced both protocol assignment and postoperative outcomes. Potential confounders include age, sex, BMI, preoperative functional status, habitual physical activity, comorbidities, and medication use. Several of these variables were not systematically available at the group level in the retrospective dataset and could therefore not be incorporated into adjusted analyses. Given the small final sample size, a multivariable regression including multiple covariates would also carry a substantial risk of model overfitting. Residual confounding must therefore be considered when interpreting between-group comparisons. Complete baseline characteristics were not available in a group-stratified format, preventing assessment and statistical adjustment for potentially relevant baseline imbalances in age, sex, BMI, diagnosis, preoperative function, and muscle strength. This limitation is particularly relevant given the non-randomized allocation and should be considered when interpreting between-group comparisons. External validity is further limited by the single-center setting, small sample size, age eligibility criteria, BMI < 30 kg/m^2^, and the exclusion of patients with contralateral THA, which may limit generalizability to broader THA populations. Incomplete data at some follow-up time points also prevented fully detailed summaries of all outcomes across groups and visits. Third, adherence to the home-based programme was assessed using patient-reported diaries rather than objective monitoring, potentially introducing recall and reporting bias. Furthermore, adherence to home rehabilitation was not objectively quantified, and potential differences in compliance between groups may have influenced the observed outcomes. Differences in actual exercise exposure cannot therefore be excluded. The study compared two experimental protocols with standard rehabilitation but did not include other available rehabilitation strategies or a comparison with alternative surgical approaches. Importantly, the experimental protocols differed from standard rehabilitation not only in the muscle group emphasized, but also in the overall number of prescribed exercises. Therefore, the present design cannot distinguish a muscle-specific effect from a potential effect of greater exercise exposure. Any between-group differences must consequently be interpreted as effects of the overall rehabilitation strategy rather than as evidence of selective muscle-targeting effects. Future studies should match the total exercise volume across groups to isolate the contribution of muscle-specific targeting. Furthermore, detailed procedural information regarding dynamometric assessment, including patient positioning, the number of repeated trials, and rest intervals between measurements, could not be reliably reconstructed from the retrospective records. This limits the reproducibility of the muscle-strength assessment protocol and should be considered when interpreting the dynamometric findings. The generalizability of these findings is also limited by the single-center design and the highly selected population undergoing AMIS THA. The principal comparative assessments were limited to the first postoperative month; therefore, delayed differences in muscle strength or function that might emerge at 6–12 months could not be evaluated. In addition, three out of the 29 initially enrolled patients did not complete the study assessments (10.3%), including two patients from the initially smaller SP2 group. Although the reasons for exclusion were not clearly attributable to the rehabilitation protocol, attrition further reduced the effective sample size and precision of the between-group estimates. The retrospective design also limited the availability of detailed session-level information required for complete exercise-intervention reporting, including exact rest intervals, exercise-specific intensity, and actual session duration. In addition, the retrospective dataset did not allow for reliable reconstruction of the complete outcome-specific pattern of missing observations or the exact sample contributing to every longitudinal model. This limits the transparency of the missing-data assessment and represents an additional limitation of the statistical analysis. Moreover, the assessment of multiple outcomes without a prospectively prespecified primary endpoint or formal multiplicity adjustment further increases the risk of chance findings. Incomplete data at some follow-up time points and the inability to reliably reconstruct complete observation-level descriptive summaries for every outcome, group, and assessment time point limited the granularity of numerical reporting. The graphical results should therefore be interpreted within the exploratory nature of the present retrospective dataset. Complete baseline characteristics were not available in a reliably reconstructable group-stratified format. Consequently, comparability between rehabilitation groups at the baseline could not be established, and potentially relevant differences in demographic characteristics, diagnosis, comorbidities, preoperative function, or muscle strength could not be adequately assessed or controlled. Residual baseline imbalance and confounding therefore cannot be excluded and substantially limit the interpretation of between-group comparisons. Finally, the study did not comprehensively assess other clinically important outcomes, including return to daily and recreational activities, pain during specific activities, patient satisfaction, and longer-term maintenance of functional recovery. These findings should therefore be considered preliminary and hypothesis-generating. Because all patients underwent THA followed by structured postoperative rehabilitation, the observed improvements over time cannot be attributed specifically to the rehabilitation protocols and likely reflect the combined effects of surgery, natural postoperative recovery, perioperative care, and rehabilitation. Future studies should use prospective randomized designs, adequately powered samples, standardized exercise volumes, objective adherence monitoring, and stratified baseline data. Longer follow-up and the assessment of return to activity, satisfaction, and clinically meaningful changes in patient-reported outcomes will also be necessary. Such studies may determine not only whether targeted flexor or abductor rehabilitation provides an overall advantage, but also whether specific patient subgroups—defined by age, preoperative weakness, functional demands, or baseline gait impairment—are more likely to benefit from an individualized muscle-specific programme following AMIS THA.

## 5. Conclusions

All three rehabilitation groups showed postoperative improvement following AMIS THA, but neither the flexor-focused nor the abductor-focused intensified protocol demonstrated superiority over standard rehabilitation. Given the small sample size, absence of an a priori sample-size calculation, non-randomized allocation, and short follow-up, these findings should be considered preliminary and hypothesis-generating. The present results do not support replacing standard rehabilitation with either targeted protocol in routine clinical practice. Importantly, because the targeted protocols differed from standard rehabilitation in both muscle emphasis and overall exercise volume, the present study could not determine whether any potential between-group effect was attributable to muscle-specific targeting or to greater exercise exposure. Adequately powered randomized controlled trials with longer follow-up are required to determine whether muscle-specific rehabilitation provides clinically meaningful benefits after AMIS THA.

## Figures and Tables

**Figure 1:**
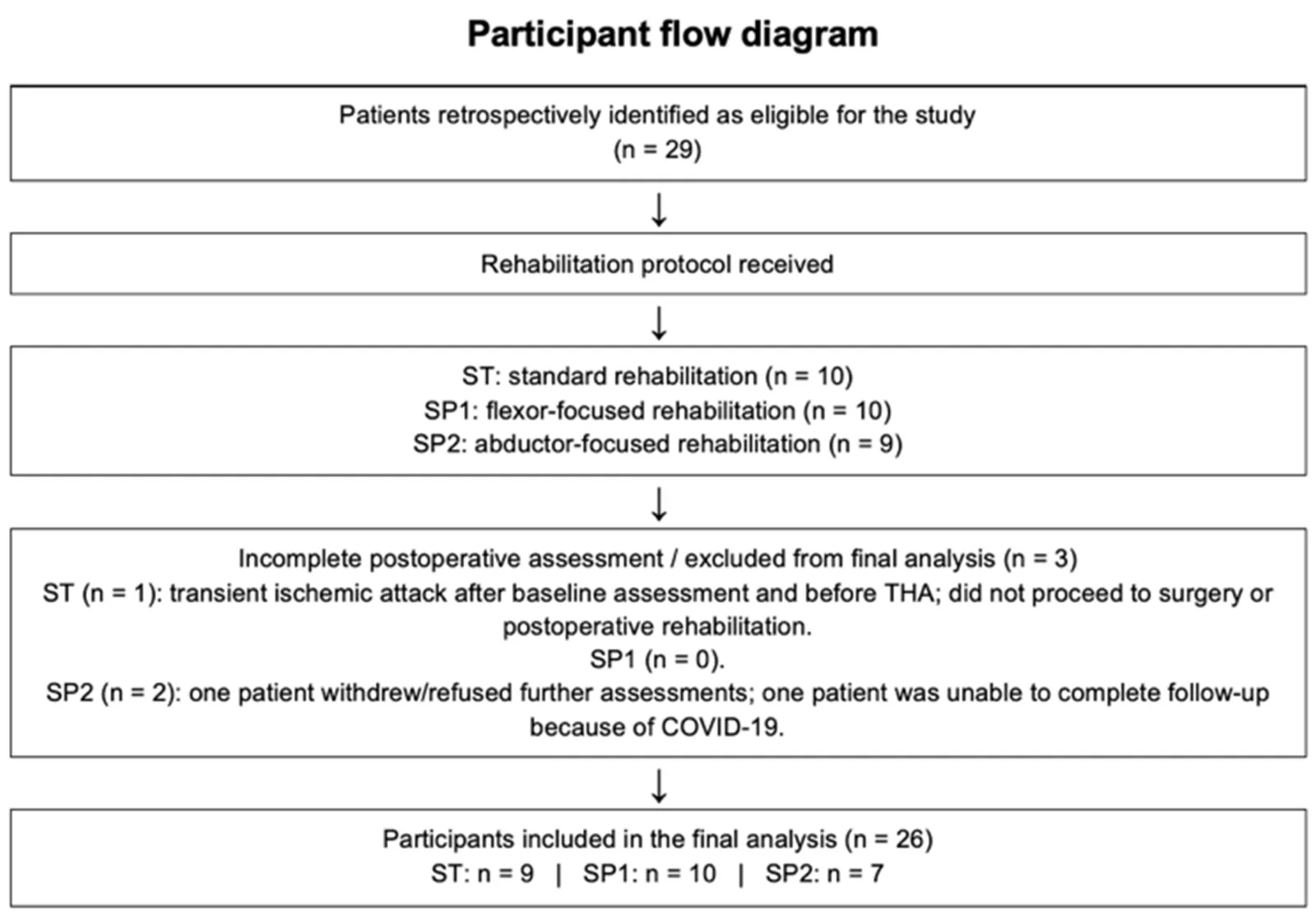
Participant flow through the retrospective study. ST, standard rehabilitation; SP1, flexor-focused rehabilitation; SP2, abductor-focused rehabilitation; THA, total hip arthroplasty.

**Figure 2:**
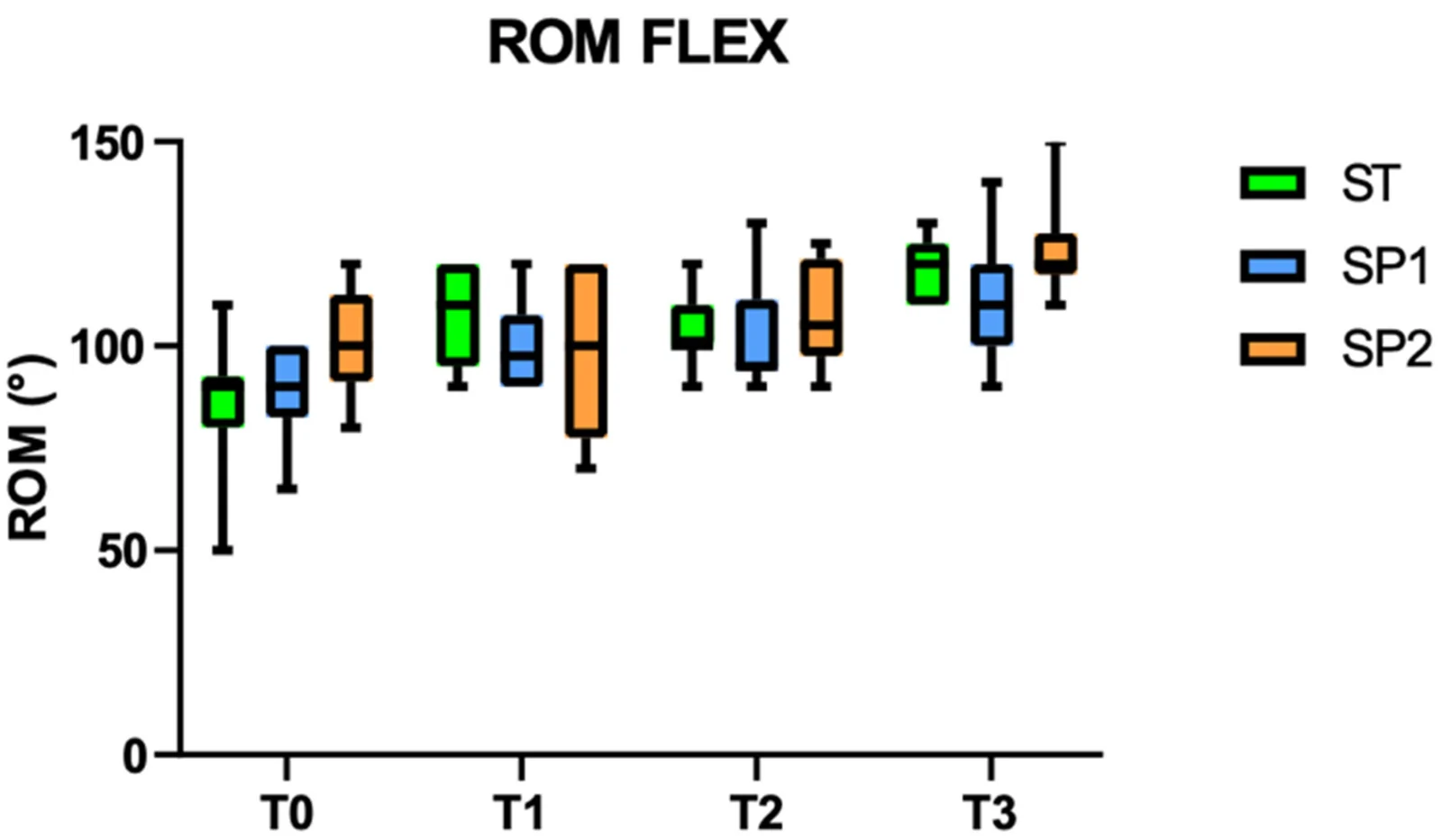
Hip flexion range of motion (ROM). ST, standard rehabilitation; SP1, hip flexor-focused rehabilitation; SP2, hip abductor-focused rehabilitation. Data are presented as median, interquartile range, and minimum–maximum values.

**Figure 3:**
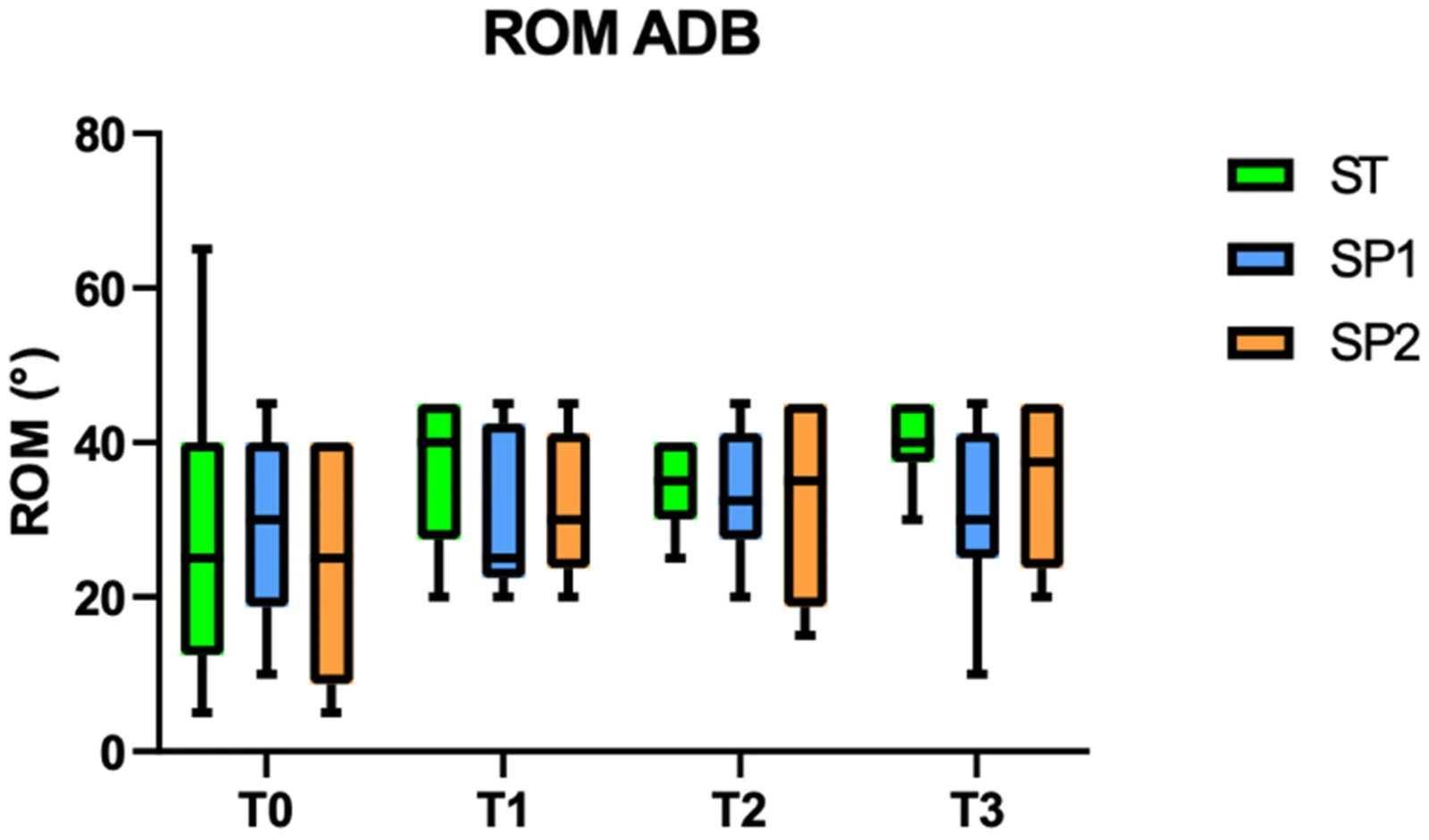
Hip abduction range of motion (ROM). ST, standard rehabilitation; SP1, hip flexor-focused rehabilitation; SP2, hip abductor-focused rehabilitation. Data are presented as median, interquartile range, and minimum–maximum values.

**Figure 4:**
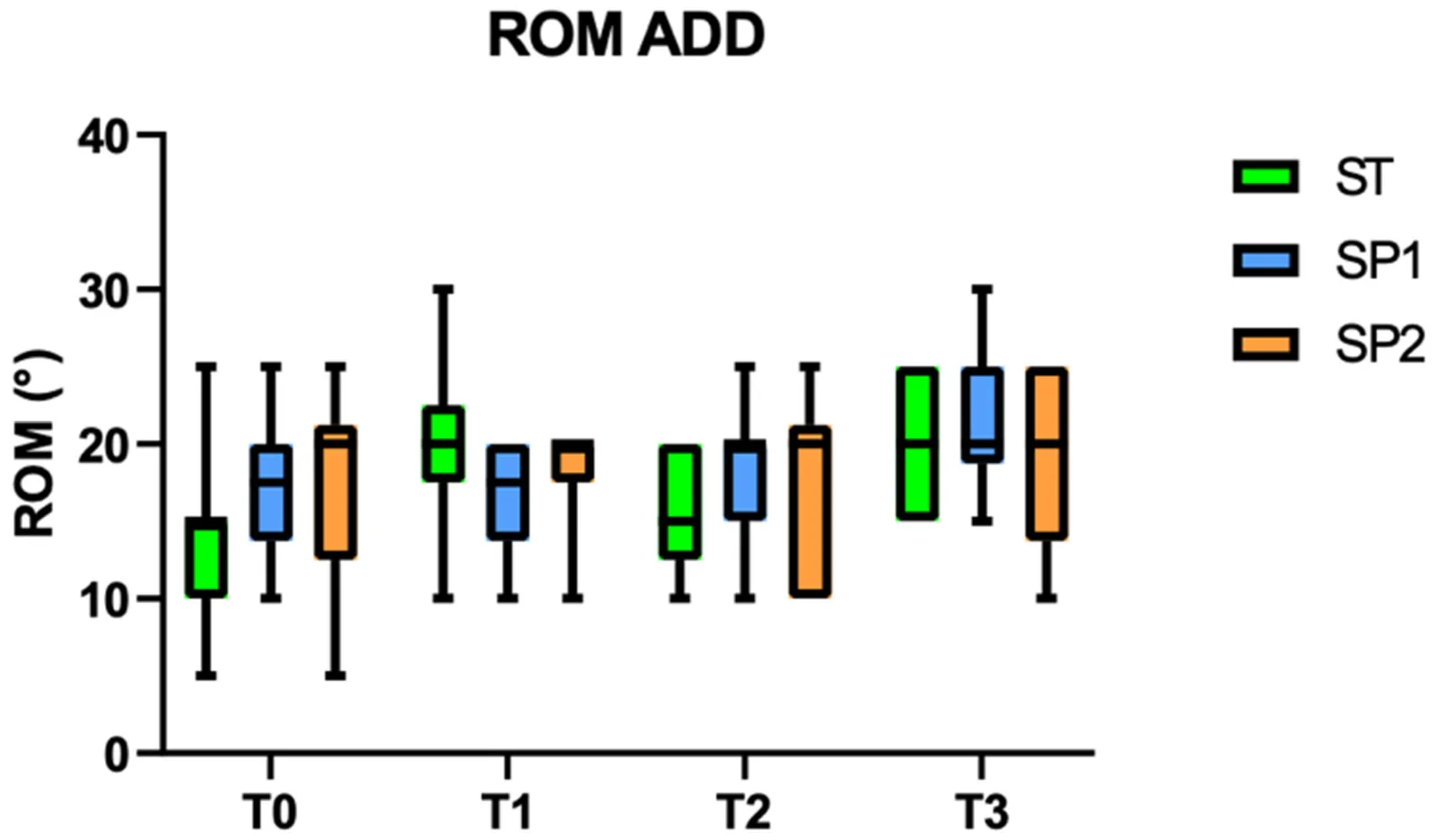
Hip adduction range of motion (ROM). ST, standard rehabilitation; SP1, hip flexor-focused rehabilitation; SP2, hip abductor-focused rehabilitation. Data are presented as median, interquartile range, and minimum–maximum values.

**Figure 5:**
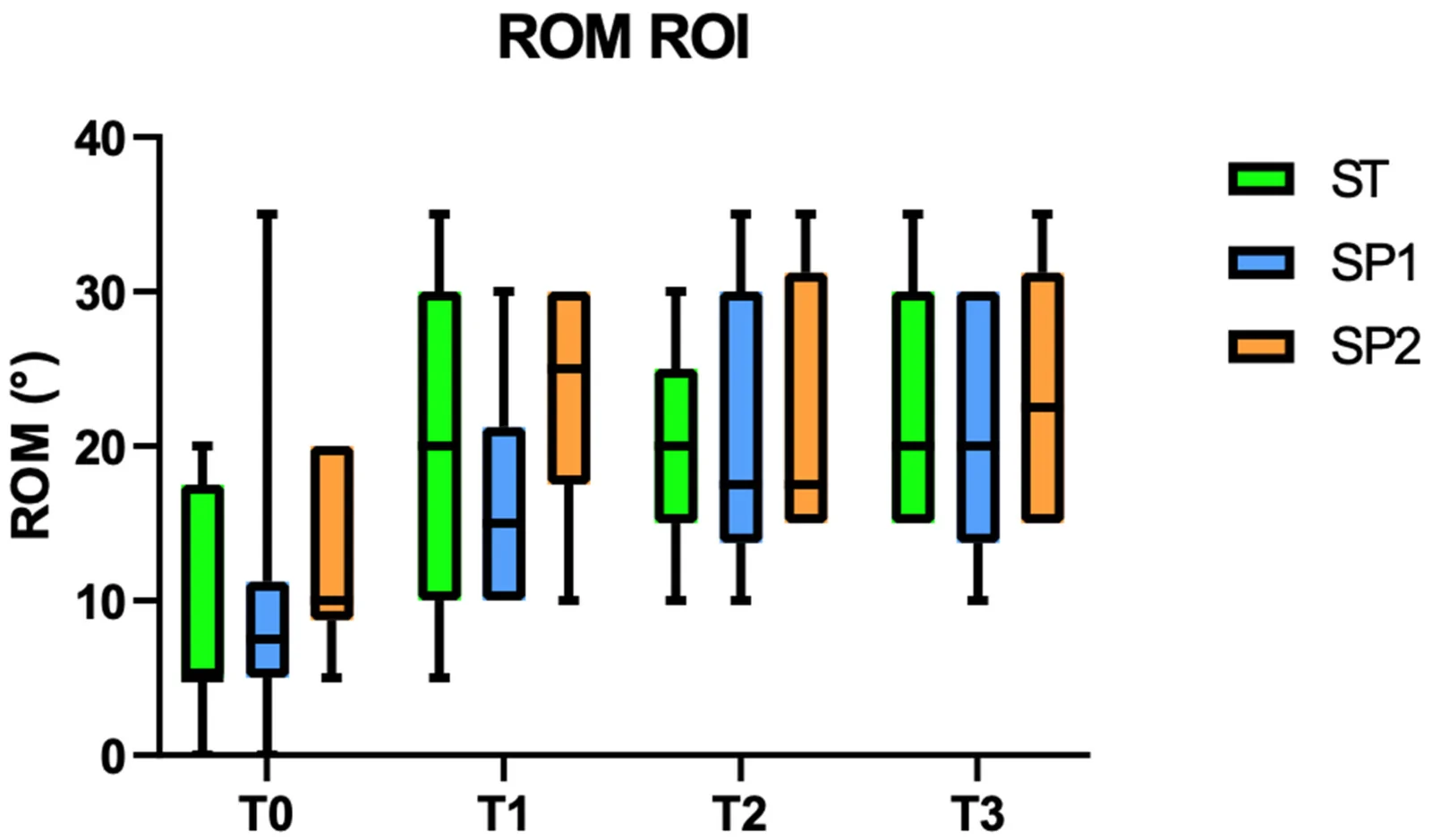
Hip internal rotation range of motion (ROM). ST, standard rehabilitation; SP1, hip flexor-focused rehabilitation; SP2, hip abductor-focused rehabilitation. Data are presented as median, interquartile range, and minimum–maximum values.

**Figure 6:**
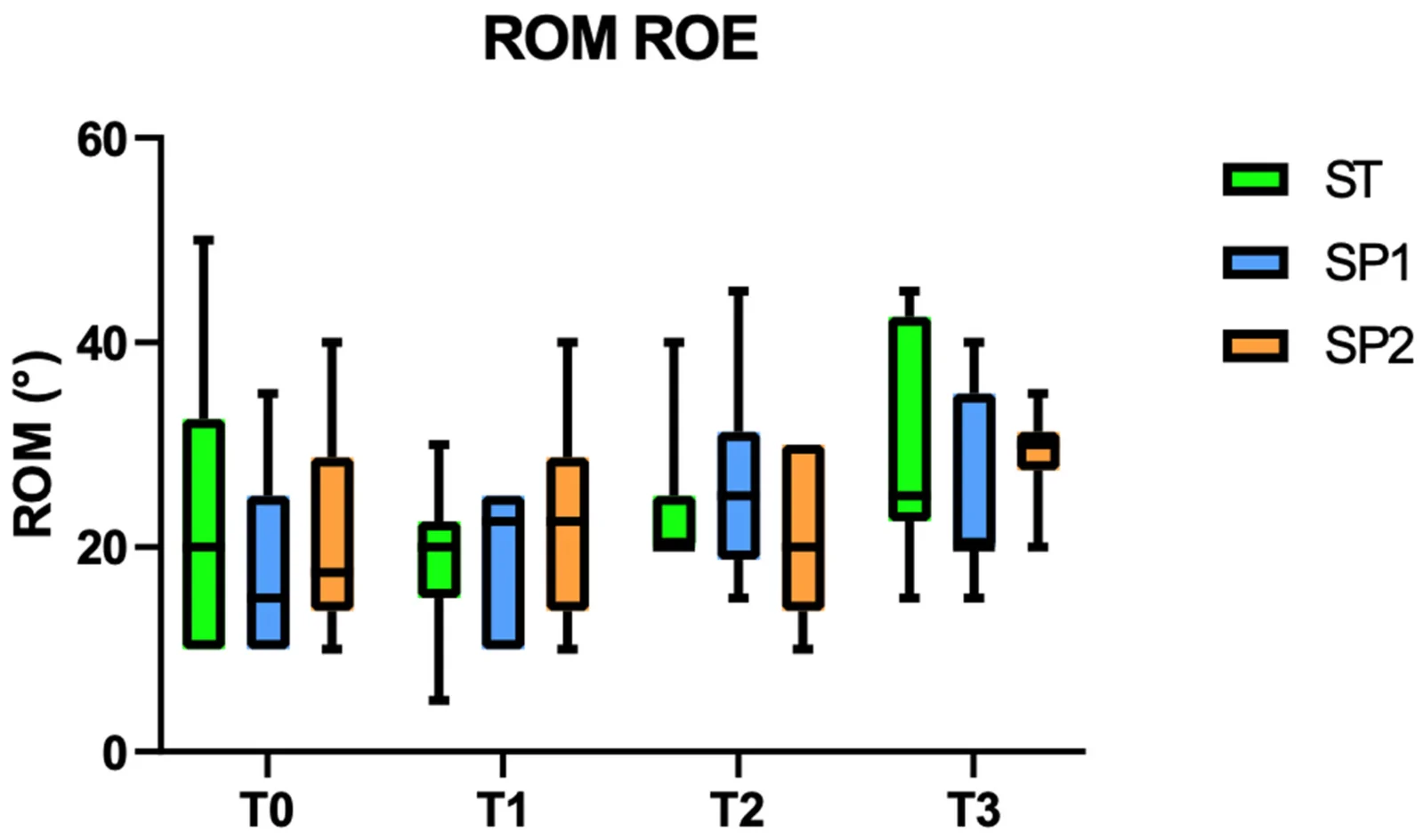
Hip external rotation range of motion (ROM). ST, standard rehabilitation; SP1, hip flexor-focused rehabilitation; SP2, hip abductor-focused rehabilitation. Data are presented as median, interquartile range, and minimum–maximum values.

**Figure 7:**
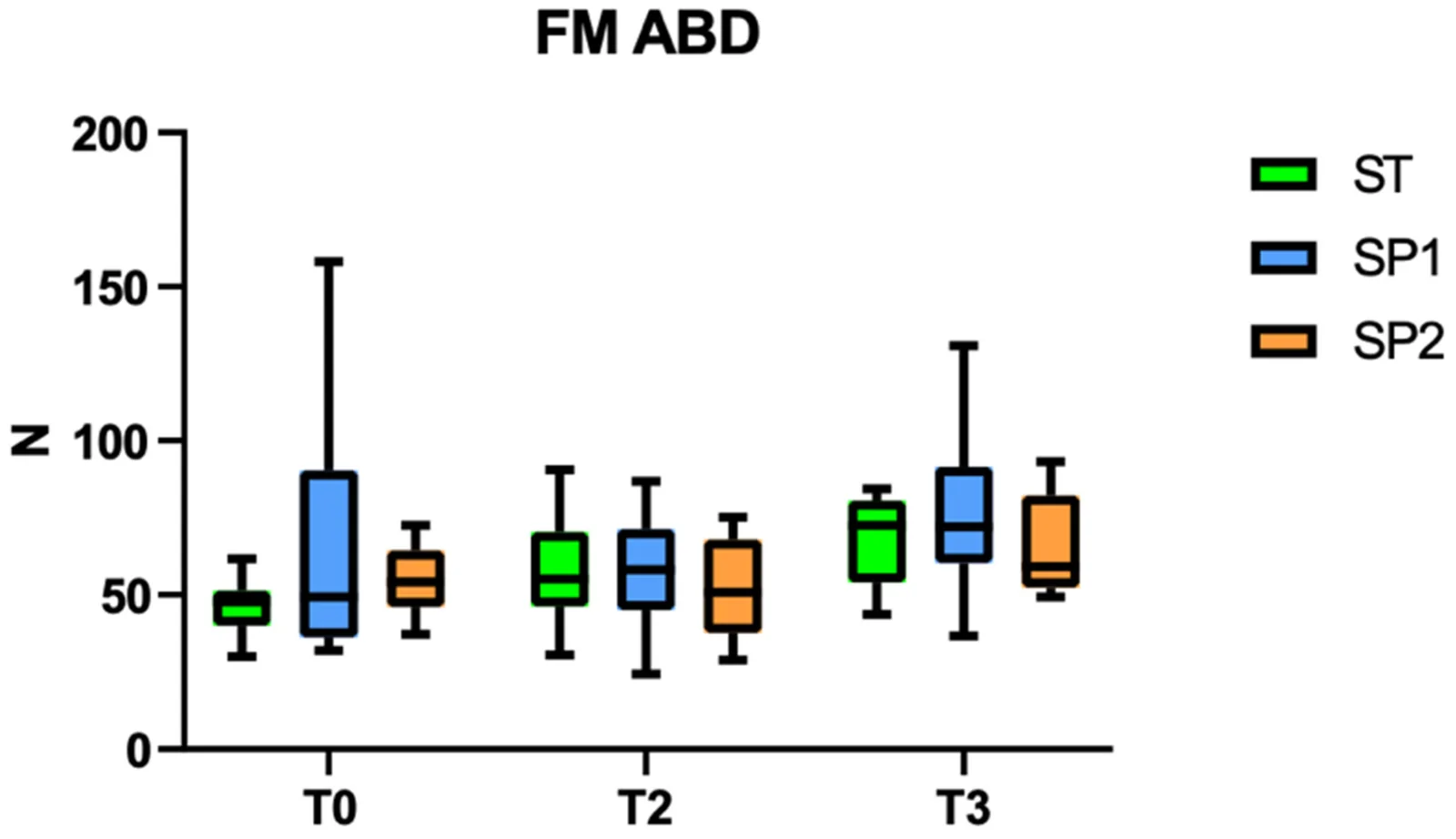
Hip abductor muscle strength. ST, standard rehabilitation; SP1, hip flexor-focused rehabilitation; SP2, hip abductor-focused rehabilitation. Data are presented as median, interquartile range, and minimum–maximum values.

**Figure 8:**
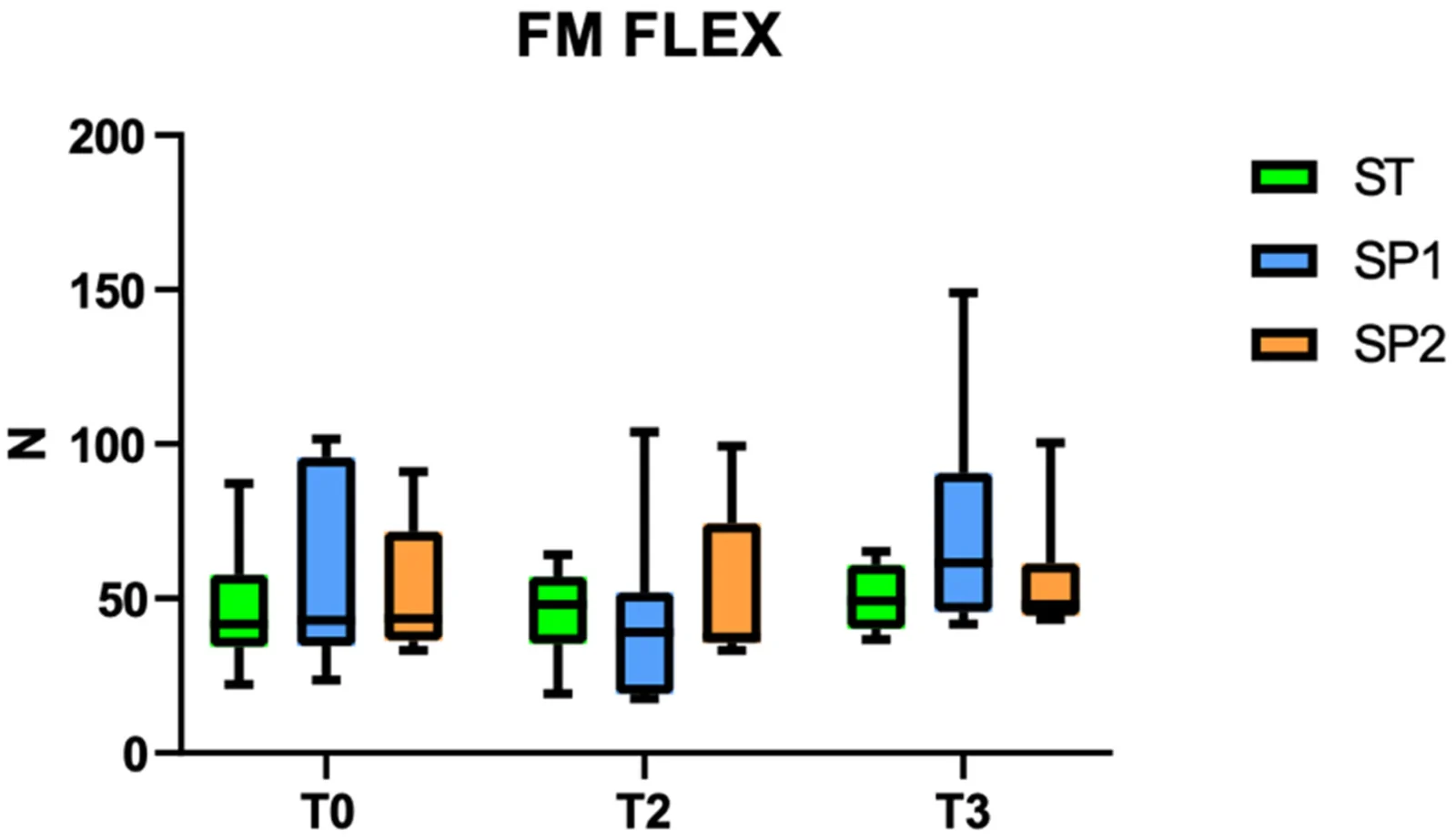
Hip flexor muscle strength. ST, standard rehabilitation; SP1, hip flexor-focused rehabilitation; SP2, hip abductor-focused rehabilitation. Data are presented as median, interquartile range, and minimum–maximum values.

**Figure 9:**
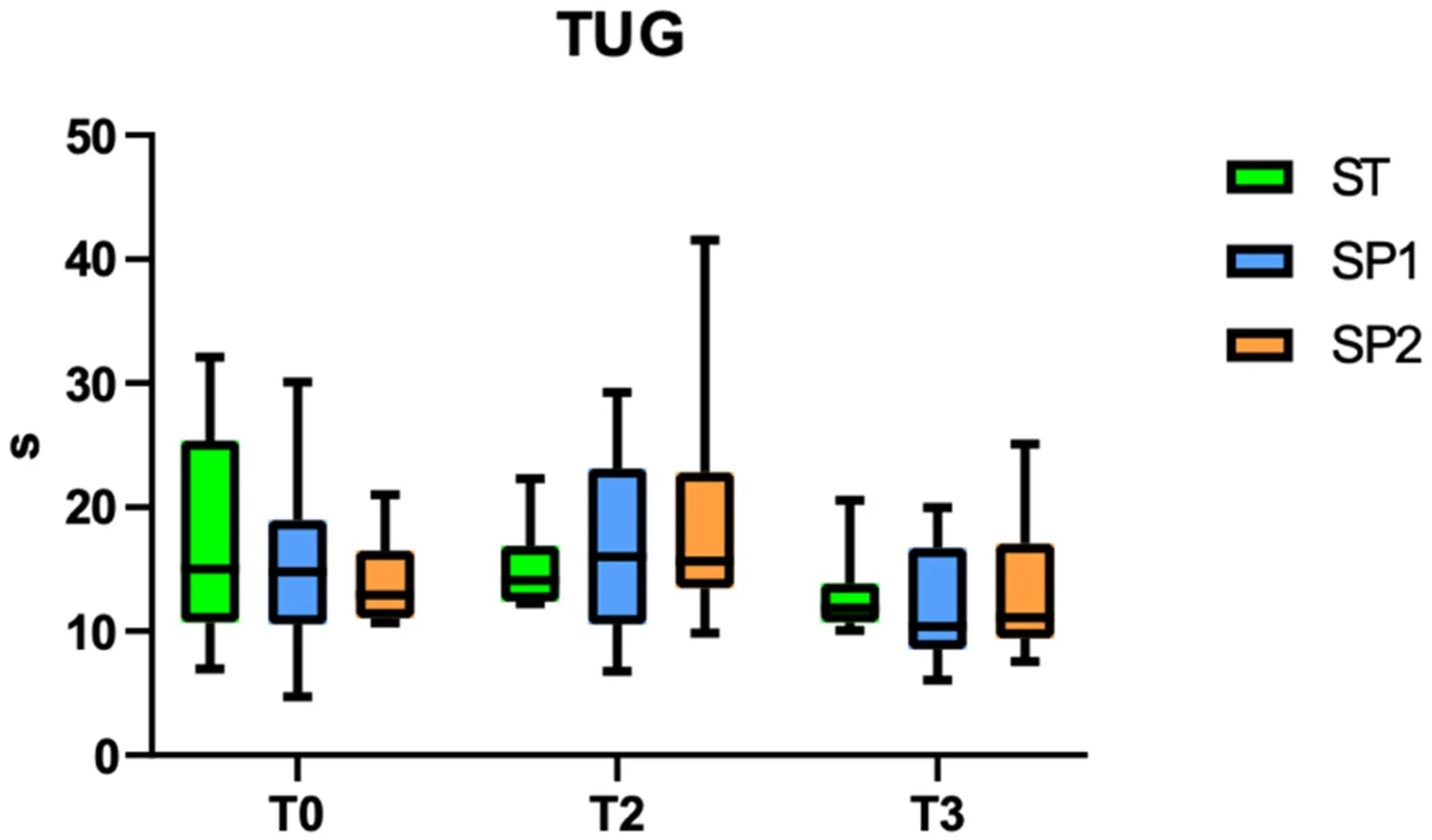
Timed Up and Go (TUG) test parameters. ST, standard rehabilitation; SP1, hip flexor-focused rehabilitation; SP2, hip abductor-focused rehabilitation. Data are presented as median, interquartile range, and minimum–maximum values.

**Figure 10:**
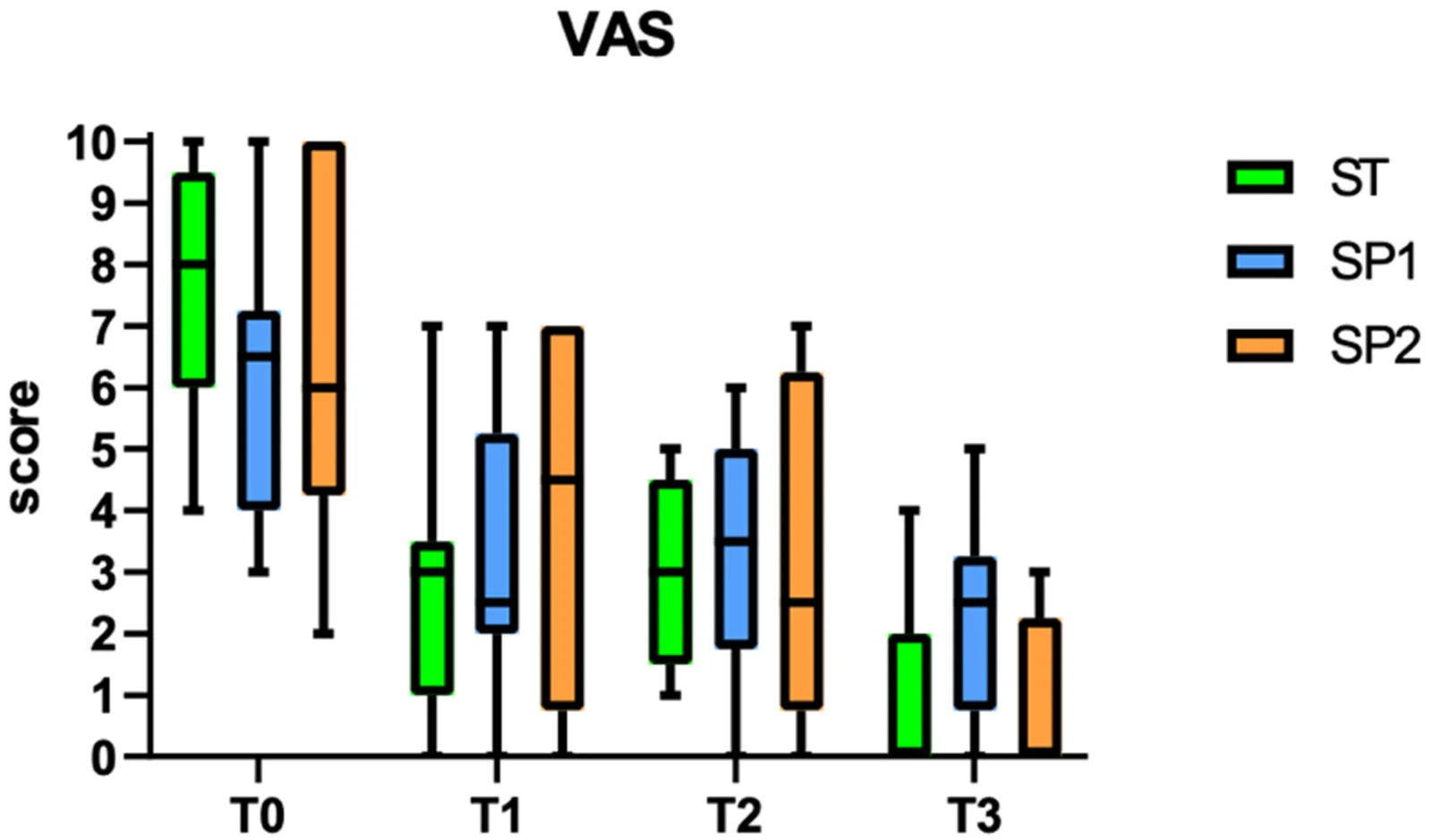
Visual Analogue Scale (VAS) test parameters. ST, standard rehabilitation; SP1, hip flexor-focused rehabilitation; SP2, hip abductor-focused rehabilitation. Data are presented as median, interquartile range, and minimum–maximum values.

**Figure 11:**
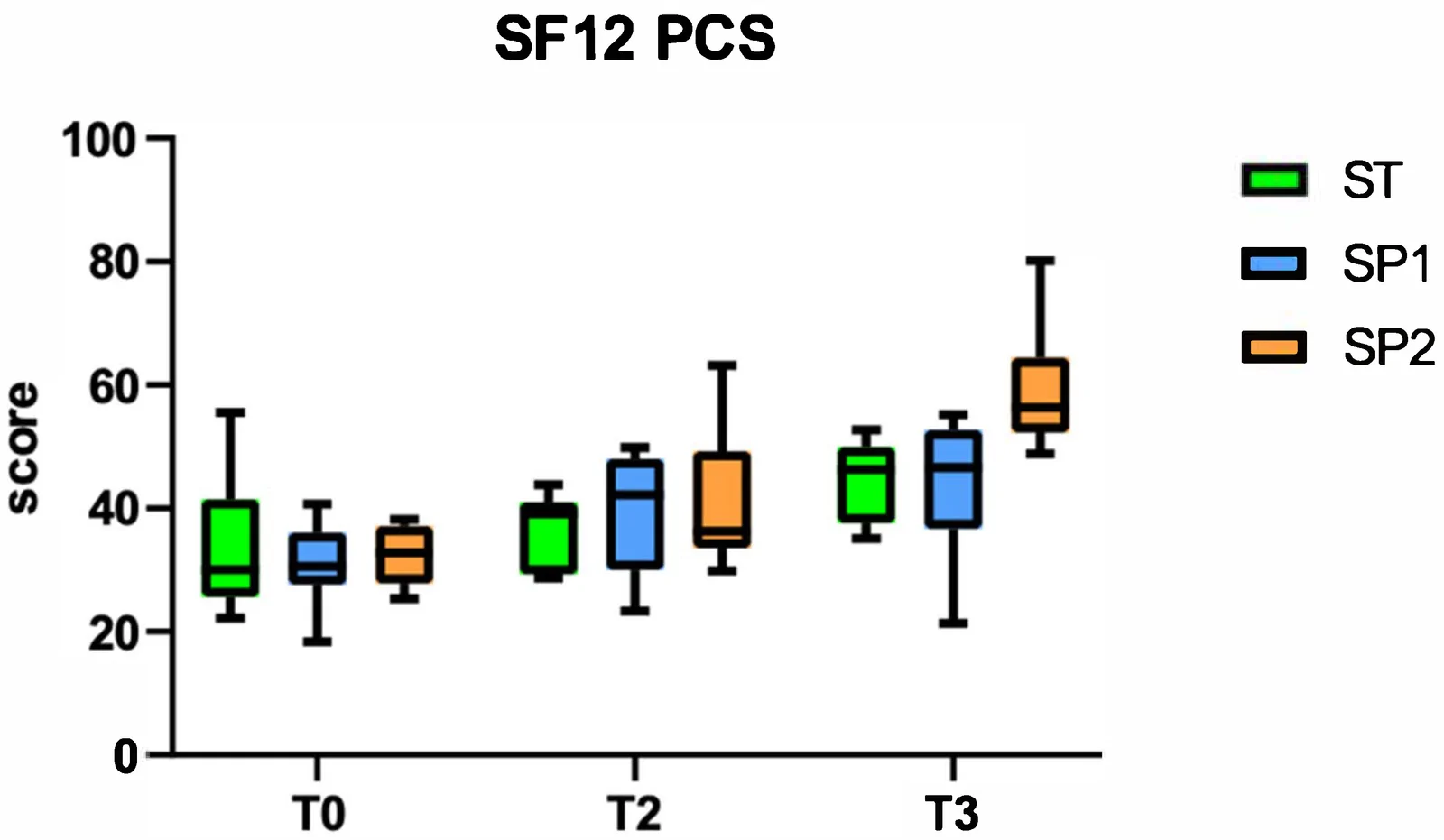
PCS-12 (Physical Component Summary) parameters. ST, standard rehabilitation; SP1, hip flexor-focused rehabilitation; SP2, hip abductor-focused rehabilitation. Data are presented as median, interquartile range, and minimum–maximum values.

**Figure 12:**
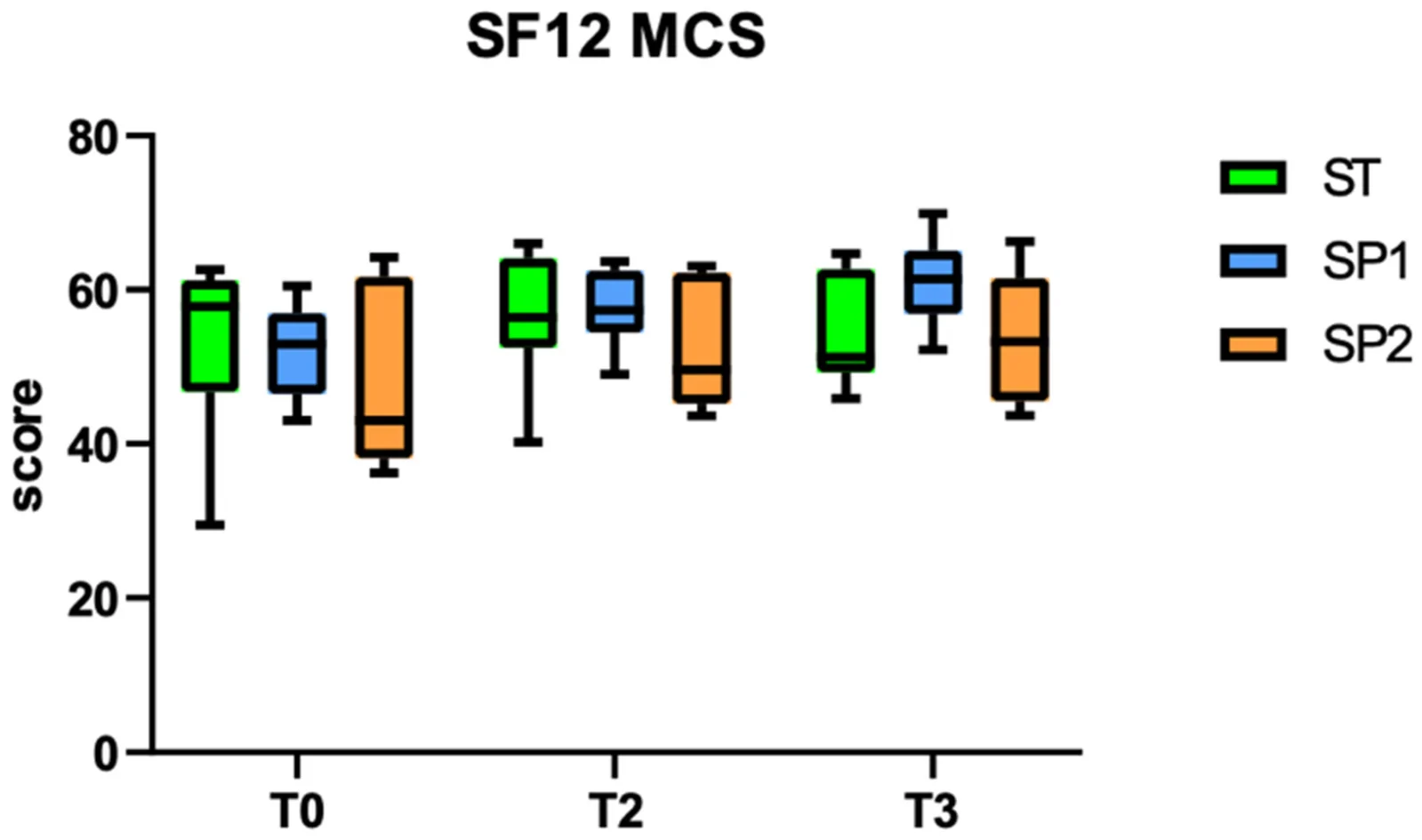
MCS-12 (Mental Component Summary) parameters. ST, standard rehabilitation; SP1, hip flexor-focused rehabilitation; SP2, hip abductor-focused rehabilitation. Data are presented as median, interquartile range, and minimum–maximum values.

**Figure 13:**
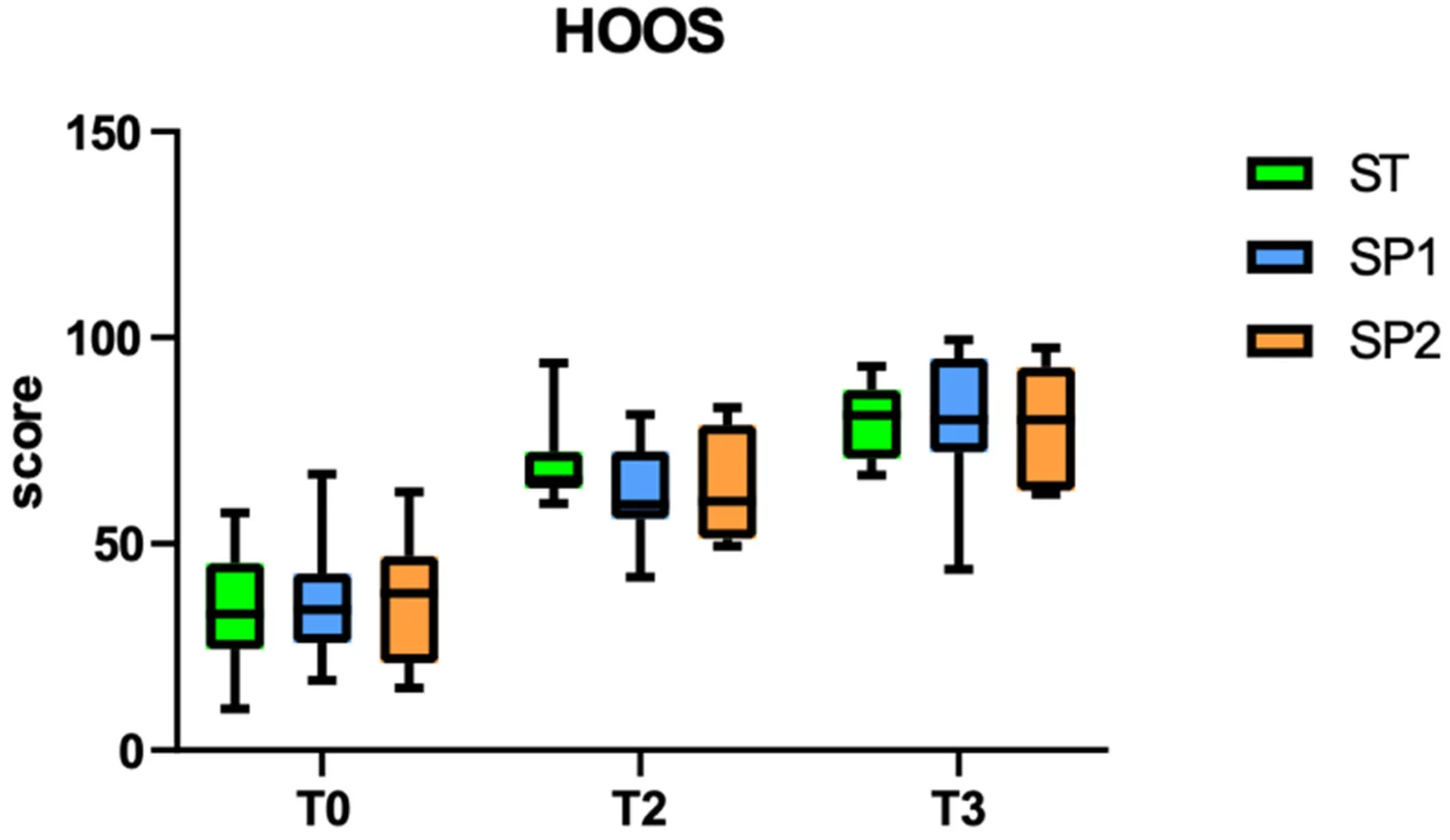
HOOS questionnaire parameters. ST, standard rehabilitation; SP1, hip flexor-focused rehabilitation; SP2, hip abductor-focused rehabilitation. Data are presented as median, interquartile range, and minimum–maximum values.

**Figure 14:**
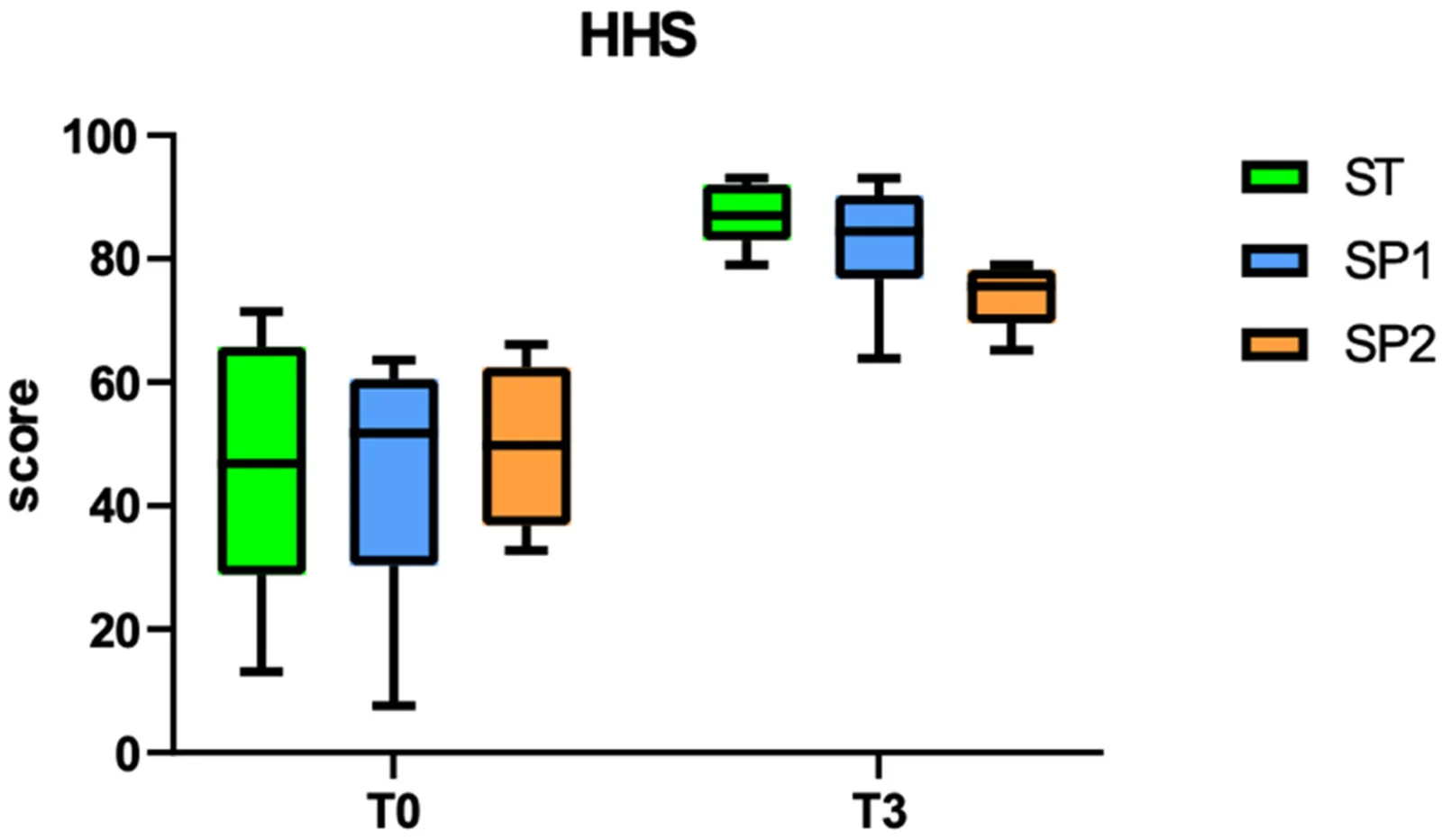
HHS questionnaire parameters. ST, standard rehabilitation; SP1, hip flexor-focused rehabilitation; SP2, hip abductor-focused rehabilitation. Data are presented as median, interquartile range, and minimum–maximum values.

**Table 1:** Rehabilitation protocols of the three different groups.

First Group (ST)	Second Group (SP1)	Third Group (SP2)
2 quadriceps strengthening exercises	2 quadriceps strengthening exercises	2 quadriceps strengthening exercises
2 hip flexor strengthening exercises	7 hip flexor strengthening exercises	2 hip flexor strengthening exercises
1 abductor muscle strengthening exercise	1 hamstring strengthening exercise	1 hamstring strengthening exercise
Bridge exercise	1 abductor muscle strengthening exercise	6 abductor muscle strengthening exercises
1 ankle plantar flexors strengthening exercise	Bridge exercise	Bridge exercise
1 weight shifting exercise	1 ankle plantar flexors strengthening exercise	1 ankle plantar flexors strengthening exercise
Semisquat	1 weight shifting exercise	1 weight shifting exercise
Stationary bike (only with orthopedic approval)	Semisquat	Semisquat
	Stationary bike (only with orthopedic approval)	Stationary bike (only with orthopedic approval)

**Table 2:** Baseline demographic and clinical characteristics of the study population (N = 29). Abbreviations: BMI = body mass index.

Age Group (Years)	Sex	n	Mean Age (Years)	Mean BMI
50–59	Male	2	50	24.8
	Female	2	56	21.6
60–69	Male	6	64	28.1
	Female	5	65	25.2
70–80	Male	6	77	25.8
	Female	8	76	22.8

## Data Availability

The datasets generated and/or analyzed during the current study are available from the corresponding author on reasonable request, subject to institutional, ethical, and patient-privacy restrictions.

## Abbreviations

The following abbreviations are used in this manuscript:

ADL	Activities of Daily Living
AMIS	Anterior Minimally Invasive Surgery
ERAS	Enhanced Recovery After Surgery
FM ABD	Abductor Muscle Strength
FM FLEX	Flexor Muscle Strength
HHS	Harris Hip Score
HOOS	Hip Disability and Osteoarthritis Outcome Score
MCS-12	Mental Component Summary
PCS-12	Physical Component Summary
ROM	Range of Motion
ROM ADD	Adduction Range of Motion
ROM ABD	Abduction Range of Motion
ROM FLEX	Flexion Range of Motion
ROM ROE	External Rotation Range of Motion
ROM ROI	Internal Rotation Range of Motion
THA	Total Hip Arthroplasty
TUG	Timed Up and Go
VAS	Visual Analogue Scale
